# Supplementary material for: Establishing farm dust as a useful viral metagenomic surveillance matrix
Source: Sci Rep. 2022 Sep 29;12:16308. doi: 10.1038/s41598-022-20701-x (PMC9521564; doi:10.1038/s41598-022-20701-x)
Supplement: Supplementary file 1 — Supplementary Information 1. [file 41598_2022_20701_MOESM1_ESM.docx]

**Supplementary Materials**

**Supplementary Figures**

**Figure S1.** An overview of virus family prevalence in chicken feces and farm dust samples. Associated metadata is shown at the top panel. The color intensity of the heatmap (bottom panel) is determined by number of contigs with minimum length of 300 nt, at least 70% identity and an e-value threshold of 1x10^-10^ when comparing with closest reference in our database. Sample order is assorted by farm ID, sampling time point. Time point is an arbitrary number. Samples were collected at the same time and place when both farm ID and time point match.

**Figure S2.** An overview of picornavirus genera prevalence in chicken feces and farm dust samples. Associated metadata is shown at the top panel. The color intensity of the heatmap (bottom panel) is determined by number of contigs with minimum length of 300 nt, at least 70% identity and an e-value threshold of 1x10^-10^ when comparing with closest reference in our database. Sample order is assorted by farm ID, sampling time point. Time point is an arbitrary number. Samples were collected at the same time and place when both farm ID and time point match.

**Supplementary Table Legends**

**Table S1.** A list of corresponding BioProject, BioSample and SRA accession numbers for all samples.

**Table S2.** A list of corresponding GenBank accession numbers for sequences used in phylogenetic analyses.
